# Interdiffusion at the Molybdenum–TEOS Interface in Flat and Nanostructured Systems during Graphene CVD

**DOI:** 10.1021/acs.langmuir.6c00096

**Published:** 2026-06-24

**Authors:** S. Zappalà, M. Scuderi, S. Mirabella, D. Costantino, A.L. Pellegrino, A. Sciuto, G. Compagnini, V. Privitera, E. Rimini, G. D’Arrigo

**Affiliations:** † Consiglio Nazionale delle Ricerche - Istituto per la Microelettronica e Microsistemi (CNR-IMM), Z.I. VIII Strada, 5, Catania 95121, Italy; ‡ Department of Chemistry, University of Catania, Viale A. Doria 6, Catania 95123, Italy; § Department of Physics and Astronomy, University of Catania, Via S. Sofia, 64, Catania 95123, Italy

## Abstract

The integration of graphene with metallic layers provides a versatile platform for hybrid materials in electronic and optical technologies. Here, we investigate interdiffusion phenomena at the molybdenum–tetraethyl orthosilicate (TEOS)–derived SiO_2_ interface during graphene growth by chemical vapor deposition (CVD). Graphene was grown on a 35 nm thick continuous sputtered molybdenum film and on nanostructured molybdenum patterns defined by electron beam lithography, both supported on a 1.6 μm thick TEOS–derived SiO_2_ layer. The samples were characterized by Rutherford backscattering spectrometry, secondary ion mass spectrometry, X-ray diffraction, and scanning and transmission electron microscopy. On the flat molybdenum film, the CVD process leads to the formation of Mo–Si and Mo–C phases and to the growth of approximately 15 graphene layers. In contrast, nanostructured molybdenum undergoes solid-state dewetting, resulting in morphological instability and the formation of only about 5 graphene layers. These results demonstrate that catalyst geometry critically influences graphene thickness: interdiffusion is predominantly unidirectional in planar films, whereas it becomes multidirectional in confined nanostructures, leading to partial carbon loss and enhanced silicon incorporation. The loss of structural homogeneity below a critical lateral dimension highlights the key role of interfacial diffusion and surface morphology in controlling graphene growth on metal–oxide substrates.

## Graphical abstract

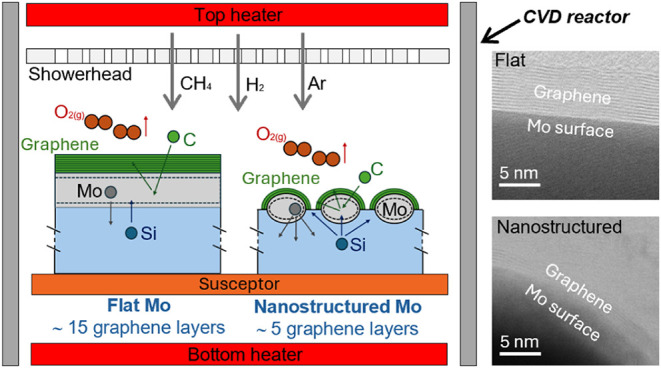

## Introduction

Graphene was first isolated from graphite in 2004 using mechanical exfoliation.^1,2^ This material is useful for research and engineering applications thanks to its electronic, thermal, and mechanical properties.^3–6^ In particular, graphene produced through Chemical Vapor Deposition (CVD) on a metal catalyst is compatible with microfabrication processing.^7,8^ As reported in literature, copper is the most common metal catalyst used for the fabrication of graphene monolayers by CVD,^9,10^ but its application in wafer-scale fabrication is difficult due to its low melting point (1083.4 °C^11^), very close to the temperature used in CVD graphene, and to its diffusion coefficient in silicon [4.7·10^3^ exp­(−0.43/kT) cm^2^/s] in the temperature range from 400 to 900 °C.^12^ Moreover, Cu can diffuse in the oxide present on silicon through the atomic substitution mechanism, and this is one of the main obstacles to the use of copper for ultralarge-scale integration (ULSI) applications.^13,14^ Furthermore, copper diffusion in silicon is enhanced in the presence of carbon, which leads to the formation of copper silicide particles.^15^ Beyond the issues related to diffusion, the use of copper as a catalyst for CVD of graphene presents additional disadvantages. Device microfabrication is difficult because of the need to pattern structures only after graphene growth and subsequent mechanical transfer to the target substrate.^16,17^ Moreover, the high difference in thermal expansion between Cu (17 μm·m^–1^·K^–1^) and Si substrates (2.6 μm·m^–1^·K^–1^) leads to the formation of wrinkles that degrades the charge carrier mobility.^18–20^ A possible solution to improve the fabrication of devices based on graphene is the use of molybdenum as a CVD catalyst.^21,22^ Indeed, molybdenum offers several advantages: high melting point (2623 °C), low thermal expansion coefficient (4.8 μm·m^–1^·K^–1^), and a low diffusion coefficient in silicon (0.26 exp [(−2.2) eV/kT] cm^2^/s).^23,24^ Molybdenum is also compatible with device microfabrication because patterning by lithography can be performed before metal deposition and graphene growth, avoiding the additional mechanical transfer steps required when copper is employed.^25,26^

Moreover, molybdenum has been demonstrated as a suitable catalyst for graphene growth, where carbon diffusion through the metal and subsequent surface segregation lead to the formation of multilayer graphene films.^22^

It is fundamental to choose the right substrate to realize devices based on graphene grown on molybdenum. Among the available dielectric materials, tetraethyl orthosilicate (TEOS) is widely employed in the microelectronics industry as a precursor for SiO_2_ layers.^27,28^ Its extensive use in semiconductor fabrication derives from the ability to form uniform films through chemical vapor deposition processes, ensuring excellent electrical insulation and compatibility with standard microfabrication technology.^29^ In the present work, “TEOS” is used for simplicity to denote the TEOS–derived SiO_2_ layer, including in the sample nomenclature and figure labels. The adoption of substrates based on TEOS, in this work, provides a technologically relevant platform, enabling direct evaluation of graphene growth on molybdenum.^30^

Previous studies have shown that Mo thin films deposited on crystalline silicon substrates form MoSi_2_ at temperatures around 520–550 °C.^31^ Furthermore, diffusion studies in molybdenum silicides indicate a strong asymmetry between the diffusivities of the constituent elements, with silicon diffusion being several orders of magnitude faster than molybdenum diffusion, suggesting that silicide growth is primarily controlled by Si migration.^32^ In crystalline Si substrates, the atomic ordering and crystallographic orientation may therefore influence interfacial nucleation and diffusion pathways.^31^ TEOS-derived SiO_2_ layers are amorphous and therefore do not impose crystallographic constraints on diffusion processes, in contrast to crystalline silicon, where diffusion pathways can be influenced by lattice orientation.^32^ Consequently, interdiffusion in Mo/TEOS systems is mainly governed by thermally activated reactions at the interface during the CVD process.

At the same time, the elevated temperatures required for graphene growth can affect the stability of the underlying layers, highlighting the importance of optimized processing conditions to ensure reliable device integration.^33^ The aim of this work is to investigate interdiffusion phenomena at the molybdenum–TEOS interface in order to explore material compatibility for semiconductor technology and to allow the realization of scaled conductive paths through a self-aligning process.^34,35^

Graphene was grown by chemical vapor deposition (CVD) on both flat and nanostructured molybdenum substrates to evaluate the effect of catalyst morphology on growth behavior. The nanostructured sample was patterned by electron beam lithography (EBL), followed by the sputtering of a 35 nm molybdenum film, and identical CVD growth conditions were applied to both flat and patterned substrates. Graphene deposition was carried out in low-pressure CVD (LPCVD) at 1020 °C for 450 s. The resulting samples were characterized by Rutherford backscattering spectrometry (RBS), secondary ion mass spectrometry (SIMS), scanning and transmission electron microscopy (SEM, TEM, STEM), X-ray diffraction (XRD), and selected area electron diffraction (SAED).

## Materials and Methods

The substrates used for this work consist of silicon wafers (100) covered by a 1.6 μm tetraethyl orthosilicate (TEOS)-based SiO_2_, deposited through Plasma-Enhanced Chemical Vapor Deposition (PECVD).

### Electron Beam Lithography (EBL)

Molybdenum micro- and nanostructures were fabricated on the TEOS/Si substrate by electron beam lithography (EBL). To this aim, a positive resist (PolyMethylMethacrylate, PMMA-AR-P 670.02, All Resist) 270 nm thick was deposited by spin coating, using a Spin Coater Model HP8 by Suss MicroTec. A virtual mask with a nanopattern was realized using a CAD program, consisting of a 4 × 4 matrix with different geometries: four-pointed stars with a length of 6 μm and nanometric tips, interspersed with circles of three different diameters, 100, 400, and 500 nm. After the e-beam lithography (with the eLINE system by Raith, at 20 kV), the resist on the sample was developed in a standard solution of MethylIsobutylKetone/Isopropyl Alcohol (MIBK/IPA) 1:3, for 3 min.

### Sputtering of Molybdenum

After lithography, molybdenum was sputtered on the sample using a Magnetron 6″ Sputtering system by Elettrorava. A metal thickness of 35 nm was obtained by sputtering for 50 s under the following conditions: RF power set point = 500 W; throttle valve = 30%; argon flux = 14 sccm; chamber pressure = 4 mTorr. The final molybdenum nanostructures were obtained through liftoff, removing the PMMA and the metal deposited out of the pattern. For this process, we used heated acetone at 60 °C and ultrasound treatment. The same sputtering deposition was performed simultaneously on a reference flat sample consisting of the same TEOS/Si substrate but without the lithography step.

### Chemical Vapor Deposition (CVD)

Graphene growth on both molybdenum nanostructures and flat molybdenum film was achieved using the AIXTRON Black Magic Pro 6″ Chemical Vapor Deposition (CVD) apparatus. The CVD process is divided into two steps in addition to the final phase, i.e., cooling. The first step was chamber purging and conditioning: Ar was introduced to condition the chamber, and after 40 s, Ar flow continued, and H_2_ was introduced. During the second step, the pyrolysis reaction and the growth of graphene occurred. The heating of the catalyst took place at about 1020 °C in a controlled atmosphere (P = 0.1 mbar). The introduction of CH_4_, the precursor of graphene, gave rise to a pyrolysis reaction, which led to the formation of carbon radicals. Once they reach the surface, carbon radicals reacted with the catalytic substrate to give graphene nucleation, releasing byproducts such as H_2_.^36^ The provided H_2_ contributed to the reaction chain that produced the growth of graphene. The amount of gases in the chamber was as follows: Total flux of gases = 1545 sccm, Ar flux = 1425 sccm → 0.092 mbar (92.2%), H_2_ flux = 60 sccm → 0.0039 mbar (3.9%), CH_4_ flux = 60 sccm → 0.0039 mbar (3.9%). At the adopted experimental conditions, in just a few seconds, the solid solubility of carbon in the Mo sample is reached due to the value of the CH_4_ flux. At the end of the process, the reactor was cooled to below 100 °C at a pressure of 0.2 mbar, and Ar flow was stopped.

### Rutherford Backscattering Spectroscopy (RBS)

The elemental composition and its depth distribution in the flat samples were investigated by Rutherford backscattering spectrometry (RBS) using a Singletron accelerator (Cockcroft–Walton type) and a 2.0 MeV incident beam of He^2+^ ions with a spot of 1 mm^2^. Backscattered helium ions at 165° were energy-analyzed using a silicon detector with an energy resolution of 15 keV. Data analysis was carried out using the SimNRA software and the procedure described in literature.^37^

### Secondary Ion Mass Spectrometry (SIMS)

Secondary ion mass spectrometry (SIMS) depth profiling and surface imaging were performed using an M6 instrument (ION-TOF). These analyses were used to detect and map the distribution of chemical species across the different layers of the samples. Accurate conversion of sputtering time into depth is essential to quantify the distribution of Mo species within the TEOS layer. Depth profiling was carried out using a Bi^+^ primary ion beam (30 keV, 45° incidence) for analysis, combined with a low-energy O^+^ beam (1 keV) for sputtering. The sputtering rate under dual-beam conditions was therefore determined following a standard approach. The erosion rate was achieved by correlating TOF-SIMS depth profiles with independent thickness measurements from TEM cross sections and crater depth calibration. For the as-deposited (nonannealed) Mo layer, the sputtering rate was 0.051 nm/s, increasing to 0.063 nm/s after annealing. Under the same conditions, the TEOS layer exhibited a higher erosion rate of 0.13 nm/s. These differences reflect variations in density, bonding configuration, and chemical modifications induced by annealing and oxygen-assisted sputtering and must be taken into account for accurate depth-scale reconstruction in multilayer systems.

### Transmission Electron Microscopy (TEM)

The morphology of the flat and structured samples was investigated using a JEOL S/TEM Cs-corrected ARM 200F for Transmission Electron Microscopy (TEM) and scanning TEM (STEM) imaging. The voltage of the electron beam was 200 kV. Selected area electron diffraction (SAED) patterns were used to distinguish the different crystalline phases present before and after graphene growth by chemical vapor deposition for both flat and nanostructured samples. Energy-dispersive X-ray spectroscopy (EDX) was performed in spectrum imaging (SI) mode using the same JEOL ARM 200F instrument, equipped with a Centurio EDX detector. Elemental maps of Mo, Si, O, and C were acquired by integrating the characteristic X-ray signals over the respective Kα emission lines within the selected spectral imaging area. Line scan profiles were extracted along the cross-sectional depth direction from the SI dataset to quantify the depth-dependent elemental distribution across the Mo/TEOS interface. The crystallographic data were obtained from the *Materials Explorer* database.^38^ SAED patterns and all EDX data were processed using the *Digital Micrograph* data analysis software.

### X-ray Diffraction (XRD)

Structural characterizations were performed through XRD analysis in grazing incidence mode (0.5°) using a SmartLab Rigaku diffractometer operating at 45 kV and 200 mA, equipped with a rotating anode of Cu Kα radiation. The acquisition employed a 0.02° incremental step.

### Scanning Electron Microscopy (SEM)

Morphological characterizations on the structured sample have been carried out with scanning electron microscopy (SEM) before and after graphene growth. The microscope was integrated into the eLINE system (Raith), which was used for electron beam lithography. For imaging, an accelerating voltage of 10 kV and a magnification of 10k× were employed. The SEM images were acquired by using a secondary electron detector.

## Results and Discussion

The flat sample, consisting of a nominal 35 nm-thick molybdenum film sputtered onto a 1.6 μm TEOS/Si substrate, was characterized both in the as-deposited state (Mo/TEOS/Si) and after graphene growth by chemical vapor deposition (CVD) (1020 °C for 450 s).

Figure 1a shows the full RBS spectra of the Mo/TEOS/Si sample before and after CVD at 1020 °C for 450 s. The energy signals corresponding to carbon (0.50 MeV), oxygen (0.70 MeV), silicon (1.13 MeV), and molybdenum (1.69 MeV) atoms on the sample surface are indicated. Signals at lower energies arise from backscattering events occurring beneath the sample surface. For clarity, the Mo signal before and after the CVD process is shown on an enlarged scale in Figure 1b. The same approach was applied to the Si signal, as reported in Figure 1c.

**Figure 1:**
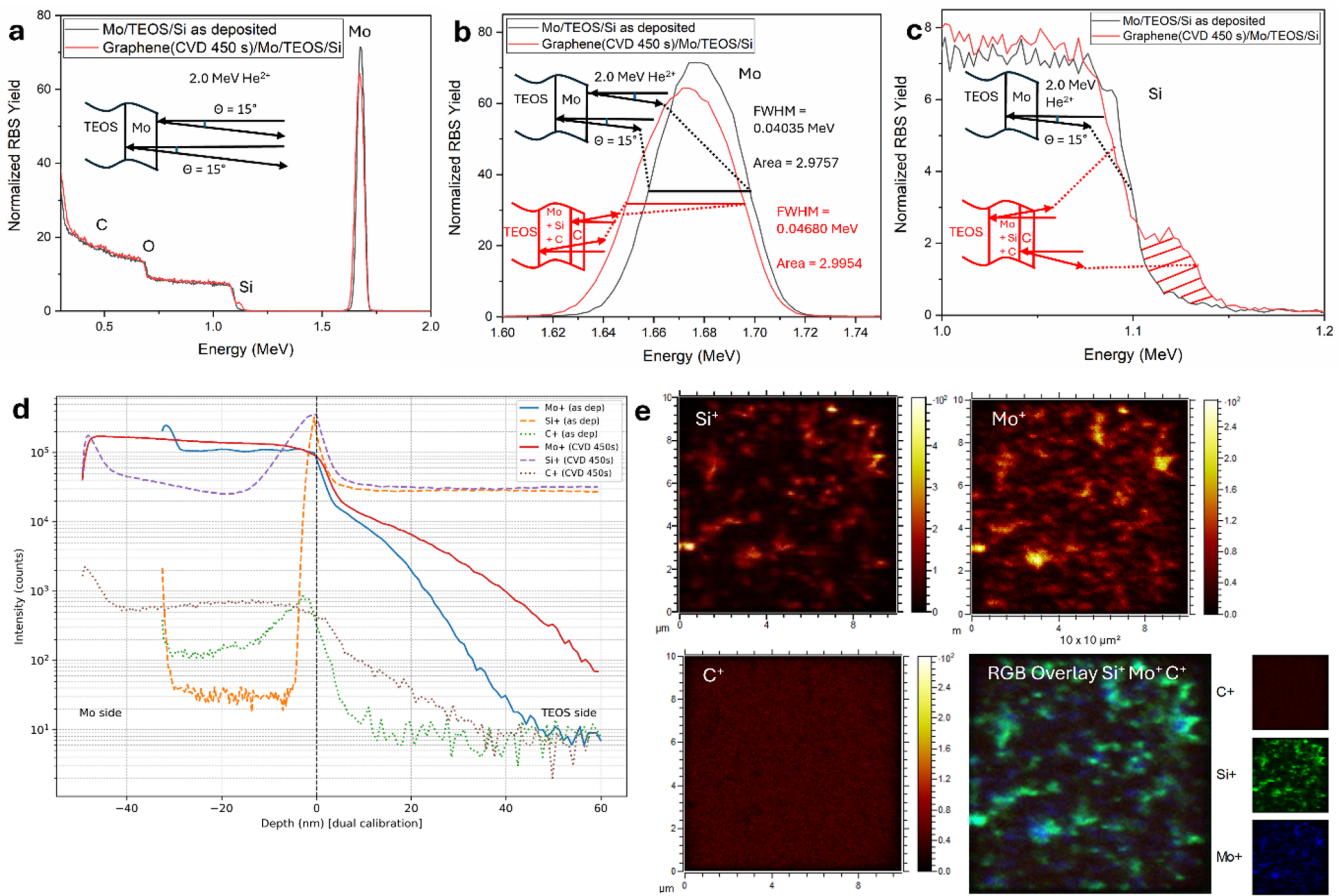
(a) RBS spectra of the as-deposited Mo/TEOS/Si (black) and after CVD at 1020 °C for 450 s (red). The main elemental signals correspond to C (0.50 MeV), O (0.70 MeV), Si (1.13 MeV), and Mo (1.69 MeV). (b, c) RBS Mo and Si signals, respectively, for the same two samples shown in (a). (d) SIMS depth profiling of Si, Mo, and C for the same two samples described in (a). (e) SIMS surface images of Si, Mo, and C and an RGB overlay image of the three elements on the surface after CVD.

The Mo spectra before and after CVD differ in peak height, full width at half-maximum (fwhm), position of the leading edge, and slope of the trailing edge. These variations arise from the combined effects of silicon and carbon diffusion into the molybdenum film and graphene formation on the sample surface. The shift of the Mo leading edge toward lower energies is attributed to the energy loss of the incident helium beam as it traverses the graphene layer. This energy loss corresponds to an areal density of 5.4 × 10^16^ C atoms/cm^2^, which, assuming a graphene areal density of 3.16 × 10^15^ atoms/cm^2^ per layer, corresponds to approximately 15 graphene layers, in agreement with TEM analysis.

The integrated area (total counts) of the as-deposited Mo spectrum corresponds to an areal density of 2.1 × 10^17^ atoms/cm^2^, as obtained from SimNRa simulations. A consistent value is also derived from the measured fwhm (0.04035 MeV) using the procedure described in the literature.^37^ Assuming a molybdenum density of 10.28 g/cm^3^, this corresponds to a film thickness of approximately 35 nm.

After the CVD process, the decrease in peak height and the increase in fwhm of the Mo signal are attributed to the additional energy loss of helium ions due to the presence of diffused silicon and carbon within the film. The Si peak shifts toward higher energies, with its leading edge corresponding to silicon located at the sample surface. The integrated area of the Si signal (dashed region in Figure 1c), from the surface to the SiO_2_ interface, corresponds to an areal density of 2.6 × 10^16^ Si atoms/cm^2^.

To estimate the content of carbon present in the Mo layer, we computed the energy loss of the helium beam in traversing a layer with the amount of Mo and Si atoms obtained by Figure 1b and 1c. The difference between the measured fwhm of the Mo energy signal and that previously computed is associated with the presence of carbon that is estimated in the range of 3.5–6.5 × 10^16^ C atoms/cm^2^. The large uncertainty is due to the low stopping power of carbon and to the Z^2^ Rutherford cross-section dependence, where Z is the atomic number.

As a last point, we consider the difference in the slope (from 90% to 10%) of the front, 24.6 keV, and of the back edge, 29.8 keV, of the Mo signal. The difference results in a layer 10–15 nm thick at the Mo–TEOS interface characterized by a decreasing concentration of Mo.

Figure 1d shows the depth-calibrated SIMS profiles of Si, Mo, and C for the as-deposited Mo/TEOS/Si and for the graphene/Mo/TEOS/Si sample state, respectively. In the as-deposited sample, the silicon peak at the interface with molybdenum is related to the matrix effect.^39^ The combined influence of the conductive Mo layer and the insulating TEOS induces surface charge accumulation, affecting the ionization yields of the detected species. After CVD, the shape of the silicon profile indicates the migration of Si atoms toward the surface, whereas the molybdenum profile reveals the slight diffusion of Mo atoms into TEOS, consistent with the findings obtained from the RBS analyses. Carbon is already detectable in the as-deposited sample, most likely because of chamber contamination. After CVD, the carbon signal increased, consistent with the growth of graphene. In the as-deposited sample, the carbon signal is relatively low within the Mo bulk and shows an apparent increase at the Mo/TEOS interface. However, this increase is not attributed to a real carbon enrichment but rather to matrix effects and possible charging phenomena during SIMS analysis associated with the transition from metallic Mo to the oxide TEOS matrix. Consequently, the detected carbon in the as-deposited condition is mainly of adventitious origin, and the interfacial increase should be interpreted as an analytical artifact. Overall, the carbon signal remains significantly lower than that observed in the processed sample, indicating the absence of significant carbon incorporation into the as-deposited film.

In contrast, in the processed sample, the higher carbon signal reflects the incorporation of carbon within the molybdenum layer during the CVD process. Under these conditions, carbon is not only present as a surface species but is distributed within the material. Therefore, the carbon signal cannot be interpreted as arising from a single species (e.g., C^–^ or C^+^ at mass 12), but rather as the cumulative contribution of multiple carbon-containing species. Due to matrix effects inherent to SIMS analysis, a strictly quantitative evaluation is not possible; however, the overall increase in the carbon signal provides a reliable indication of enhanced carbon incorporation after CVD.

Figure 1e shows the SIMS surface images of Si, Mo, and C and an RGB overlay image of the three elements after the CVD process. At the surface, a clear carbon signal is detected, corresponding to the graphene overlayer, while the Si and Mo signals appear nonuniform, indicating the formation of Mo–Si and Mo–C phases.

The structural characterization through XRD measurements corroborates the silicon and carbon diffusion into the molybdenum film and graphene formation on the sample surface. Indeed, the XRD pattern of the Mo/TEOS/Si sample (Figure 2a) displays peaks at 40.52°, 58.53°, and 73.54° related to the 110, 200, and 211 reflections of the Mo layer as a cubic crystalline structure (PDF card n. 00-042-1120), whereas the analysis on Mo/TEOS/Si after graphene growth (Figure 2b) shows several peaks associated with the formation of silicide and carbide phases, namely Mo_5_Si_3_, MoSi_2_, and Mo_2_C. The signal at 43.80° can be attributed to the 004 reflection of the graphene phase (PDF card n. 00-050-1082).

**Figure 2:**
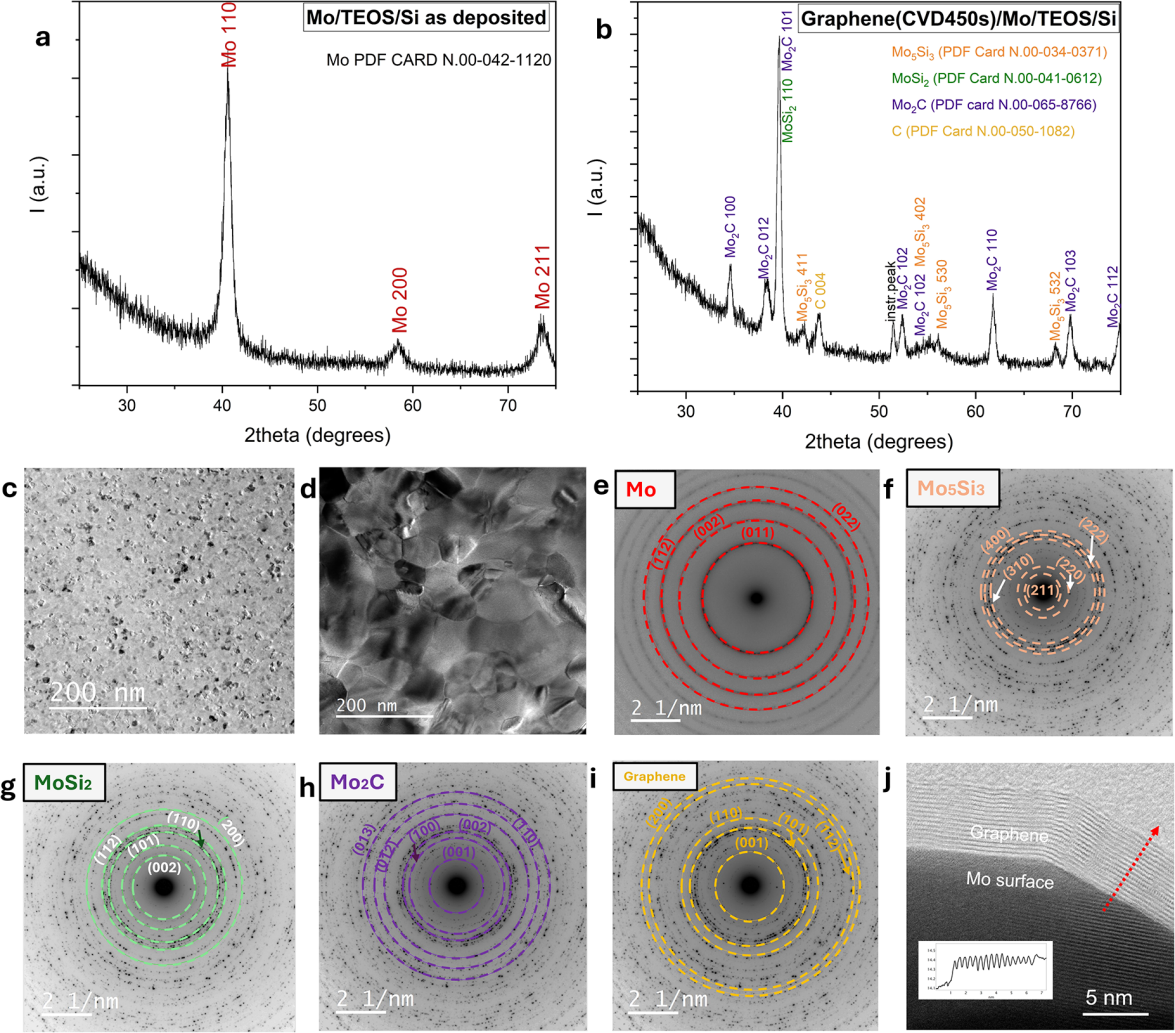
(a, b) XRD patterns of Mo/TEOS/Si before and after graphene growth by CVD at 1020 °C for 450 s. (c, d) Plan-view TEM bright-field images of Mo/TEOS/Si before and after graphene growth by CVD at 1020 °C for 450 s. (e) SAED pattern of the as-deposited Mo/TEOS/Si sample, with Mo diffraction rings indexed and highlighted in red. (f–i) SAED patterns of Graphene/Mo/TEOS/Si showing indexed diffraction rings for Mo_5_Si_3_ (pink), MoSi_2_ (green), Mo_2_C (purple), and graphitic carbon (yellow). (j) Cross-sectional STEM bright-field image of Graphene/Mo/TEOS/Si showing approximately 15 graphene layers.

TEM plan-view analyses (Figure 2c and d), on the flat sample, show a large increase of the Mo average grain size after the CVD process: from 9.5 to 114.5 nm. This increase is attributed to thermally activated grain growth during the high-temperature CVD process (1020 °C, 450 s), which promotes grain boundary migration, coarsening, partial grain coalescence, and intergranular diffusion processes.

The large area of grain boundaries during the ramp from RT to process T is responsible for faster Si migration across the Mo layer since grain boundaries act indeed as preferential diffusion pathways in polycrystalline metallic films, and their density plays a key role in the interdiffusion process. Accordingly, interdiffusion in this system depends on the combined effects of film thickness, grain size, and grain boundary density. Thinner Mo films, typically characterized by finer grains and a higher grain boundary density, are expected to enhance interdiffusion between Mo, Si, and C. Conversely, thicker films, associated with larger grains and reduced grain boundary density, exhibit lower grain-boundary-mediated diffusion and therefore a more stable catalyst morphology during the CVD process. The diffusion length of Mo in TEOS-based SiO_2_ at the temperature adopted in the present work is on the order of ∼10^–7^ cm. The structure of TEOS differs from that of SiO_2_ both in the amorphous arrangement and in the stoichiometry, thus justifying the silicon atom migration in the Mo film. Carbon, instead, is characterized by a significantly larger diffusion length in molybdenum, in the range of 600–4200 nm under the present thermal conditions.^40^ The samples were also analyzed by selected area electron diffraction (SAED), and the relative patterns are reported in Figure 2e and f–i, before and after CVD, respectively. In Figure 2c, the diffraction rings, indexed to Mo, indicate the presence of very small crystalline grains in the film, as observed in Figure 2e (TEM plan-view). After CVD (Figure 2f–i), the diffraction rings appear discontinuous and partially resolved into discrete spots. This change is associated with the significant increase in grain size observed after graphene growth (Figure 2d). The larger crystallites reduce the number of grains contributing to diffraction within the selected area, and their different crystallographic orientations lead to discrete spots rather than continuous rings. Instead of Mo, new diffraction rings indexed to Mo_5_Si_3_, MoSi_2_, Mo_2_C, and graphitic carbon are observed, indicating the formation of silicide and carbide phases during the CVD process (Figure 2f–i). Figure 2j reports a cross-sectional STEM bright-field image of the Graphene/Mo/TEOS/Si sample. The dark region corresponds to the molybdenum film, where lattice fringes associated with the (100) crystallographic planes are visible. Graphitic layers are observed emerging from the molybdenum surface, with approximately 15 graphene layers clearly discernible. The interplanar spacing extracted from the intensity profile is approximately 0.33 nm (∼3.3 Å), which corresponds to the (002) basal plane spacing of graphite reported in the literature.^41^

To spatially resolve the elemental redistribution within the Mo layer induced by the high-temperature CVD process, cross-sectional STEM-EDX analysis was performed on the flat sample, as reported in Figure 3. The HAADF image (Figure 3a) shows the Mo film resting on the TEOS substrate. On top of the Mo layer, carbon is present, originating from both the deposited graphene and the carbon used during the FIB lamella preparation.

**Figure 3:**
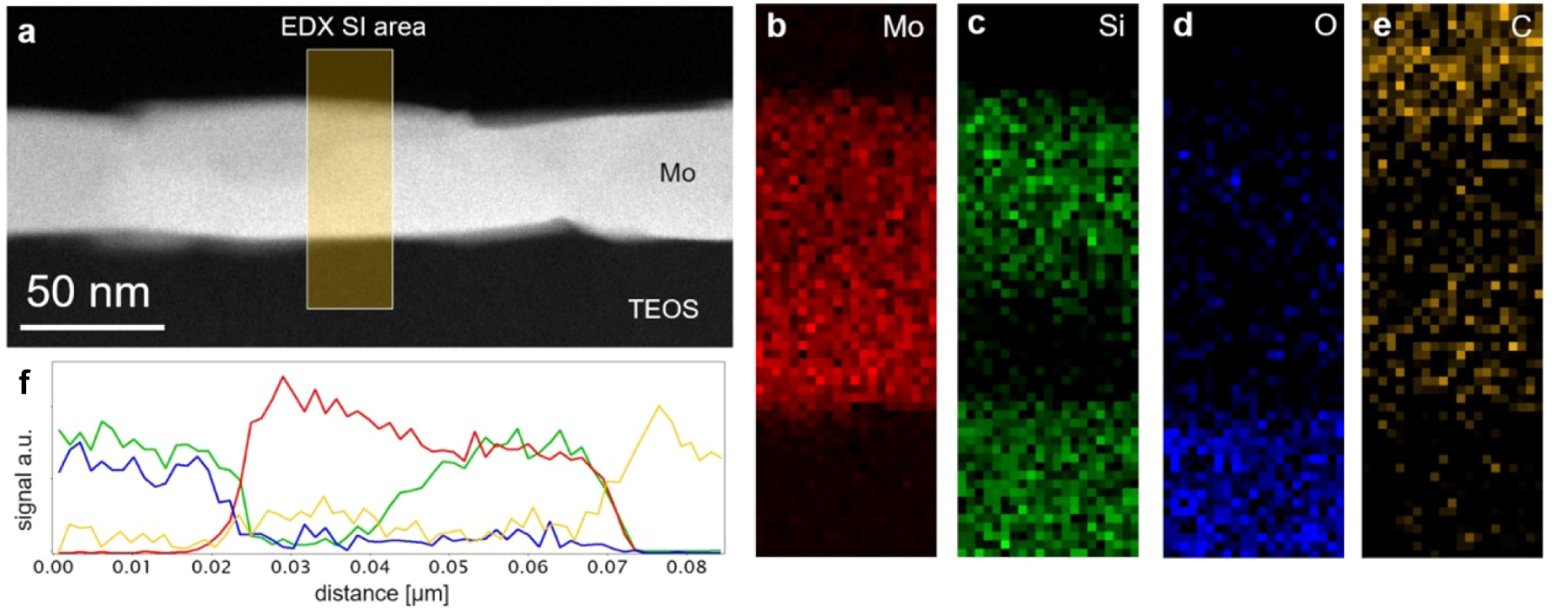
Cross-sectional STEM-EDX analysis of the flat Graphene/Mo/TEOS/Si sample after CVD at 1020 °C for 450 s. (a) HAADF-STEM image of the Mo/TEOS interface region; the yellow rectangle marks the EDX acquisition area. (b–e) Elemental maps of Mo (red), Si (green), O (blue), and C (yellow). (f) EDX line scan profiles along the depth direction (white arrow in b) for Mo (red), Si (green), O (blue), and C (yellow).

The EDX elemental maps (Figures 3b–e) and the corresponding line scan (Figure 3f) reveal a nonuniform distribution of all species across the Mo layer. The Si signal is low in the lower half of the Mo film, rises, and accumulates within the upper portion of the layer, indicating that Si has migrated upward through the Mo film during the high-temperature treatment and preferentially segregated in a subsurface region. Carbon shows a different trend: its signal is higher in the lower portion of the Mo film and decreases toward the top of the layer. This depth-dependent partitioning of Si and C is consistent with the formation of Mo–Si and Mo–C phases at distinct depths within the film, as identified by SAED (Figure 2e–i).

At the TEOS/Mo interface, the line scan reveals a localized intermixing zone: the O and Si signals exhibit finite tails extending into the bottom of the Mo layer, while the Mo signal shows a gradual onset rather than an abrupt rise when it enters the TEOS region. This indicates that chemical interdiffusion between Mo and the oxide substrate occurs during the thermal cycle, with Mo atoms penetrating the TEOS matrix and Si and O species migrating into the lowermost portion of the Mo film.

These findings are fully consistent with the RBS quantification (Figure 1b–c), which places the Si leading edge at the sample surface and determines a Si areal density of 2.6 × 10^16^ atoms/cm^2^ distributed across the Mo film, and with the depth-calibrated SIMS profiles (Figure 1d), which confirm upward Si migration across the full extent of the Mo layer during the CVD thermal cycle.

The second part of this work focuses on the nanostructured sample, in which the pattern was defined by electron beam lithography (EBL) prior to the deposition of molybdenum. The sample was characterized both before and after the CVD process. The pattern obtained by EBL consists of an array of four-pointed stars with a length of 6 μm and nanometric tips, interspersed with circles of three different nominal diameters: 100 (circle 1), 400 (circle 2), and 500 nm (circle 3). This geometry, incorporating structures with variable dimensions, was designed to investigate the effects of graphene growth at both the nanoscale and the microscale. The pattern is repeated in a 4 × 4 matrix of 1 mm elements; the total area of the structures is 25 mm^2^.

Figure 4a shows a schematic cross-section of the ion collection geometry during Mo sputtering. The substrate (TEOS) is shown in blue, while the e-beam resist (PMMA), exposed during EBL, is shown in yellow. After development, the resist exhibits nanometric cavities corresponding to the nano and microstructures described above. During sputtering, the ions follow the trajectories illustrated in the figure; the flux reaches its maximum value at the center (angle 2β) and its minimum at the edges (angle β). The collection angle at the walls is half of the central angle, resulting in reduced Mo thickness at the edges. The values of β depend on the circle diameters, measured through Scanning Electron Microscopy (SEM), and on the height of the surrounding PMMA columns. We calculated collection angles (β) for the three circles and the corresponding film thickness at their edges (t). For circle 1 (nominal diameter = 100 nm; measured diameter = 158 nm), β = 30.3° and t = 5 nm; for circle 2 (nominal diameter = 400 nm; measured diameter = 425 nm), β = 57.2° and t = 16 nm; and for circle 3 (nominal diameter = 500 nm; measured diameter = 589 nm), β = 65.4° and t = 20 nm. The collecting angle increases with increasing circle diameter. The formation of the micro- and nanostructures was confirmed by SEM, as shown in Figure 4b, which displays the three types of circles and the four-pointed stars after Mo sputtering and liftoff. The dimensions of the nanocavities are determined by the e-beam lithography mask, while their thickness is defined by the sputtering process. The actual dimensions of the nanostructures are larger than the nominal ones due to the proximity effect.^42–44^ Additional characterizations were performed after the chemical vapor deposition of graphene. Variations in nanostructure morphology were observed by SEM (Figure 4c) and plan-view TEM (Figure 4d–f). The processed film exhibited a discontinuous structure, characterized by the formation of islands. This behavior is due to a phenomenon known as solid-state dewetting, which is the spontaneous agglomeration of a thin solid film on a substrate at high temperature.^45^ Dewetting is thermodynamically driven by the reduction of the free surface energies of the film and substrate, as well as of the film–substrate interface energy. Its evolution is strongly influenced by both the film thickness and the thermal budget. In thinner films, dewetting is accelerated because the reduction of the total interfacial free energy becomes increasingly favorable as the thickness decreases, leading to earlier fragmentation of the film into isolated islands during the CVD process. Conversely, thicker molybdenum films would exhibit greater morphological stability and reduced dewetting, resulting in a more continuous catalyst layer.^46^ Accordingly, dewetting is more pronounced in the smallest nanostructures. In these regions, the reduced collection angle during sputtering leads to a thinner effective Mo film, enhancing the dewetting process, as confirmed by the higher density of smaller grains observed for the 158 nm circles compared to the 589 nm ones (Figures 4e–f). This behavior is consistent with the plan-view TEM observations (Figures 4d–f), which show a higher density of smaller Mo islands in the smallest structures.

**Figure 4:**
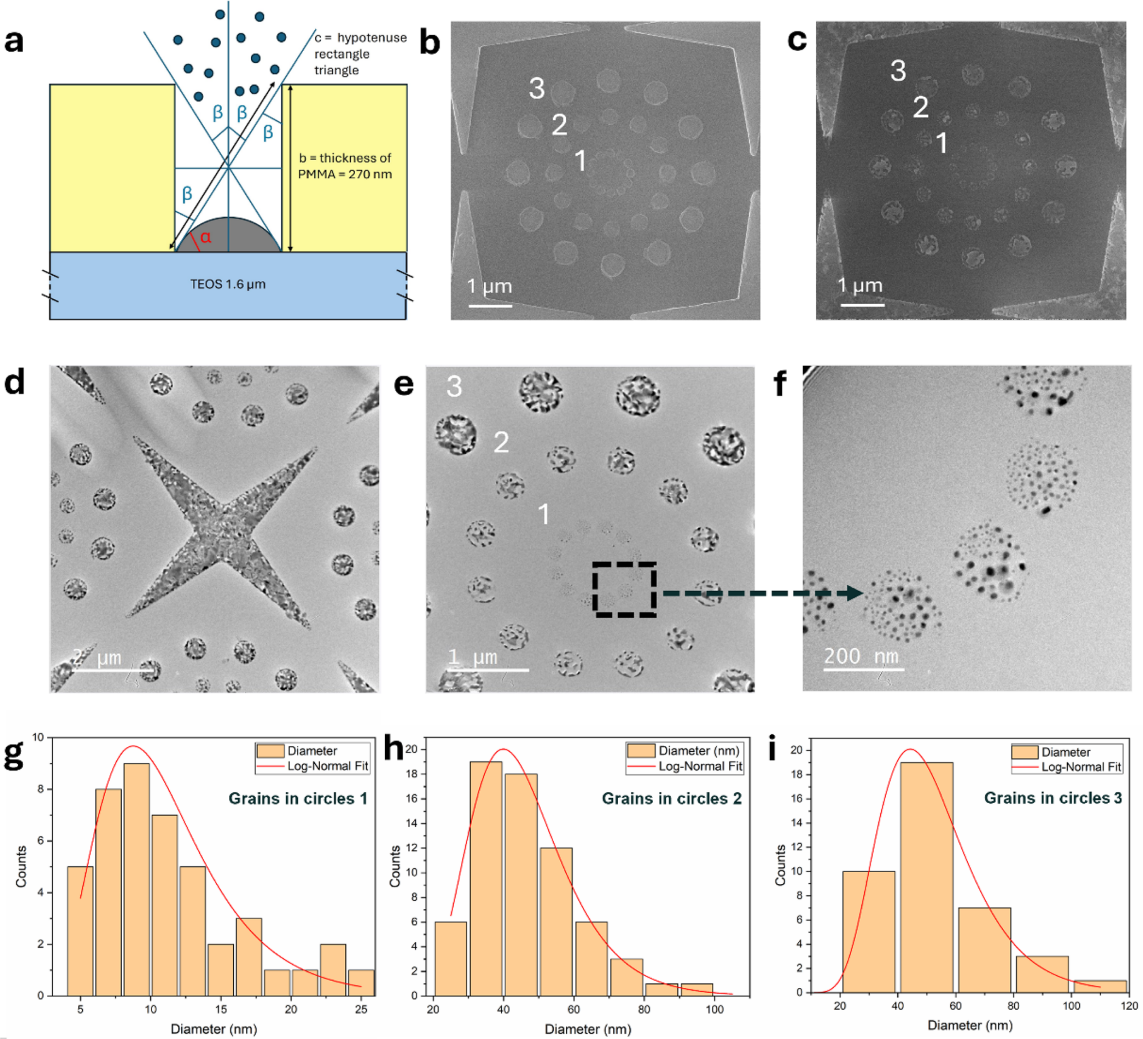
(a) Schematic cross-section of the ion collection geometry during Mo sputtering (masking effect). (b, c) SEM images of the nanostructures before and after the CVD of graphene, respectively. (d–f) Plan-view TEM images of the micro- and nanostructures after CVD (Graphene/Mo/TEOS/Si sample). (g–i) Grain size distributions within circles 1 (d_1_ = 158 nm), 2 (d_2_ = 425 nm), and 3 (d_3_ = 589 nm), respectively.

The statistical evolution of the grain size can be directly related to the geometrical parameters of the patterned structures. In particular, the diameter of the nanocircles determines the local collection angle during sputtering and, therefore, the effective Mo thickness deposited inside the cavities. Smaller circles correspond to smaller collection angles and thinner Mo layers due to the shadowing effect of the PMMA mask, establishing a direct link among pattern geometry, catalyst thickness, and dewetting behavior.

Statistical analysis of the grain size distributions within circles 1, 2, and 3 (Figure 4g–i) revealed a progressive increase in both average size and dispersion across the regions. In circle 1, the distribution is centered at smaller diameters, with a mode of approximately 8.5 nm, a median of 10.2 nm, and a mean grain size of 11 nm, indicating the formation of finer grains. In contrast, the grains inside circle 2 show a broader distribution shifted toward larger dimensions, with a median diameter around 44 nm and a mean of 47 nm. Circle 3 exhibits the largest and most polydisperse grains, with a median of 59 nm and a mean exceeding 100 nm. It should be noted that after solid-state dewetting, the lateral size of the Mo islands can largely exceed the initial film thickness. Surface diffusion promotes the agglomeration of the film into three-dimensional islands, leading to larger lateral grain dimensions than the deposited thickness. The systematic shift of the log-normal profiles thus indicates an increasing degree of coarsening and agglomeration from circle 1 to circle 3, reflecting a spatial gradient in the dewetting intensity and thermal evolution across the film. In thicker regions, the reduced dewetting and improved film continuity are expected to moderate interdiffusion during CVD. Although interdiffusion between Mo, Si, and C still occurs at high temperatures, it is likely less pronounced than in thinner regions, while a more continuous catalyst morphology may favor more uniform graphene growth.

The crystalline structures present in the nanostructured samples were investigated by SAED. Figure 5a–d show the SAED patterns acquired from the center of the star-shaped structures, where diffraction rings corresponding to Mo_5_Si_3_ (pink), MoSi_2_ (green), Mo_2_C (purple), and carbon from graphene are clearly identified by the high density of diffraction spots. In contrast, Figure 5e–h display the SAED patterns obtained from the star tip, where the same phases are detected but with a reduced number of spots. This difference can be mainly attributed to the smaller analyzed volume of material at the tip region compared to the center of the structures, which reduces the number of crystalline domains contributing to diffraction. The enhanced dewetting occurring in these regions further limits the amount of continuous Mo material available for diffraction. Possible intermixing effects in these regions are supported by the complementary SIMS, RBS, and TEM analyses discussed above. A similar trend was also observed for the three different nanocircles (SAED not shown).

**Figure 5:**
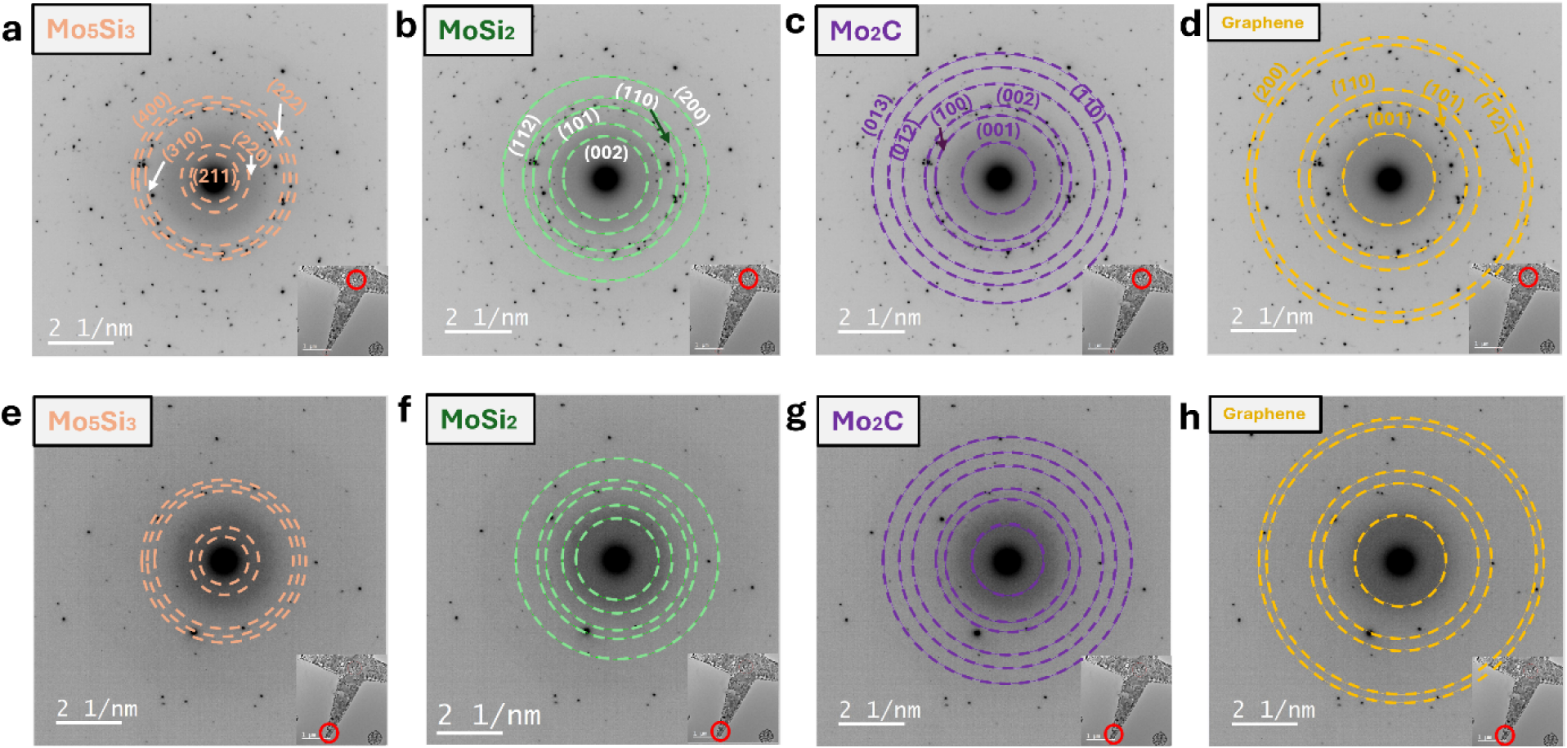
SAED patterns of the processed (CVD at 1020 °C for 450 s) nanostructured samples. (a–d) SAED from the star center with Mo_5_Si_3_, MoSi_2_, Mo_2_C, and C graphene diffraction rings, respectively. Inset: area of analysis. (e–h) SAED from the star tip with Mo_5_Si_3_, MoSi_2_, Mo_2_C, and C graphene diffraction rings, respectively. Inset: area of analysis.

Figures 6 and b schematically illustrate the CVD process for the flat (a) and nanostructured (b) samples. In both cases, molybdenum acts as a bulk catalyst for graphene growth; however, under CVD conditions, it is progressively transformed into molybdenum carbide (Mo_2_C), which actively participates in the catalytic process.^47,48^ Carbon dissolves into the metallic film and, once the solubility limit is reached (0.1 wt % for C in Mo at ∼1000 °C), it segregates at the surface, where nucleation occurs to form graphitic layers.^49^ Simultaneously, the interaction between carbon and molybdenum leads to the formation of a stable carbide phase (Mo_2_C), which exhibits a modified electronic structure and noble-metal-like catalytic behavior, enhancing CH_4_ dehydrogenation and promoting the generation of reactive carbon species.^47^ This interpretation is further supported by XRD and SAED analyses, which reveal the formation of Mo_2_C after the CVD process, accompanied by the near disappearance of metallic Mo.

**Figure 6:**
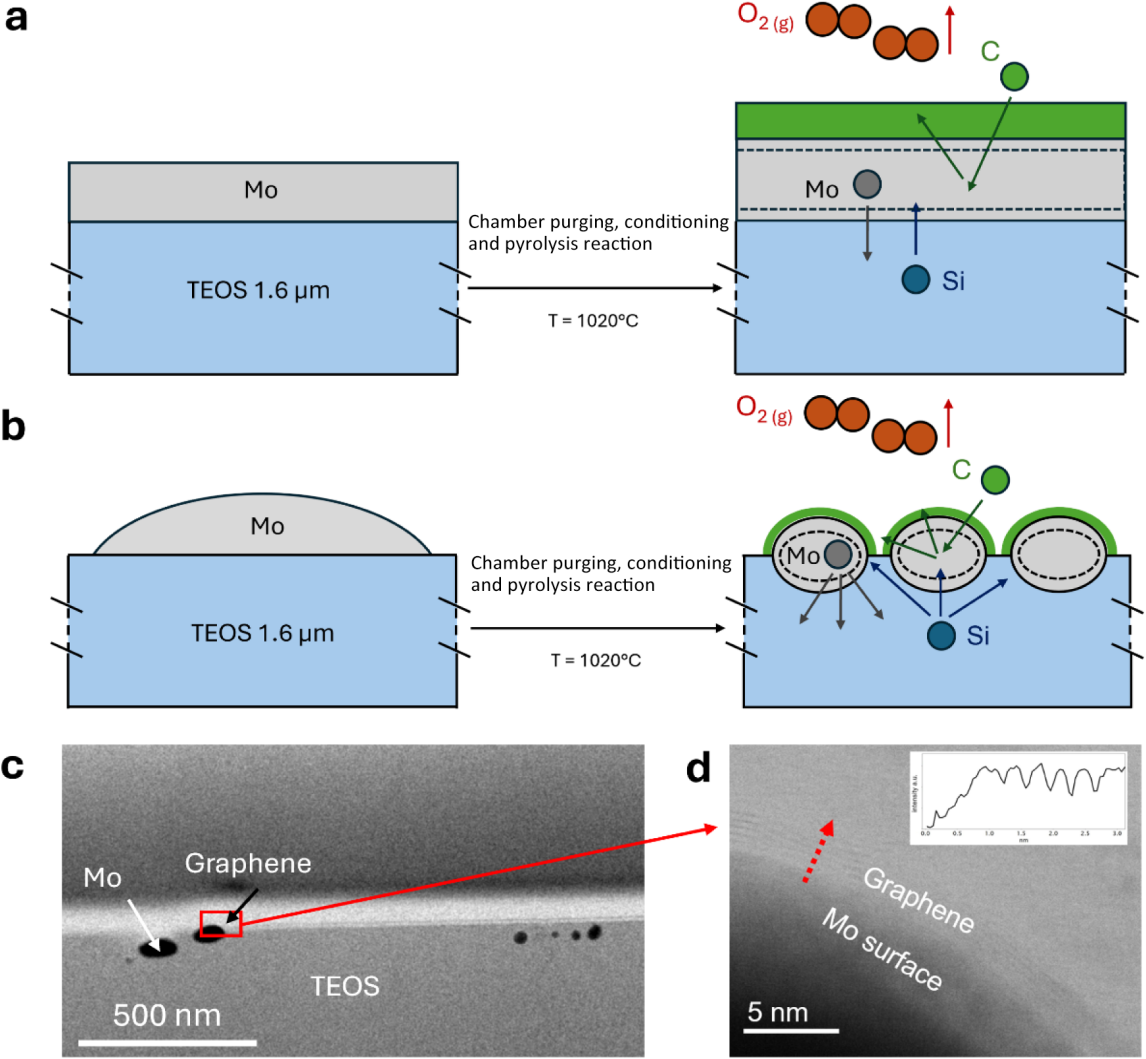
(a) Schematic illustration of CVD growth for the flat sample. (b) Schematic illustration of CVD growth for the nanostructured sample with the dewetting process, showing island and grain formation during chemical vapor deposition of graphene. (c) Cross-sectional STEM bright-field image of the nanostructured Graphene/Mo/TEOS/Si sample. (d) Cross-sectional STEM images of the grain surfaces formed within circle 2 after dewetting in the nanostructured Graphene/Mo/TEOS/Si sample.

In this framework, the catalytic mechanism can be described as a combined bulk-mediated process, where carbon diffusion and precipitation occur through the Mo_2_C matrix coupled with a surface-mediated pathway driven by Mo_2_C, which continuously supplies active carbon species for graphene growth.

At the same time, interdiffusion processes take place at both the TEOS–Mo and graphene–Mo interfaces. Part of the molybdenum diffuses into TEOS, while silicon diffuses into the molybdenum. Carbon also contributes to interdiffusion, increasing the thickness of the Mo layer on the flat sample and the dimension of the particles on the nanostructured ones. Interdiffusion of the chemical species promotes the formation of Mo–Si and Mo–C compounds. In the schematic, pure molybdenum is represented in dark gray, while the lighter gray regions correspond to molybdenum after CVD, containing diffused species, Mo–Si, and Mo–C phases (including Mo_2_C). A key difference between the two systems concerns the diffusion pathways available during the CVD process. In the flat sample, the interdiffusion processes are essentially unidirectional, mainly occurring along the thickness of the continuous Mo film. In contrast, in the nanostructured sample, the confined geometry of the catalyst induces multidirectional diffusion, which leads to a noncompensated lateral carbon flux. As a consequence, part of the carbon is redistributed within the nanostructures and does not effectively resegregate at the catalyst surface after bulk diffusion, which is required for graphene nucleation. In addition, carbon trapped within the Mo_2_C phase may further limit the amount of carbon available for surface segregation, reinforcing this effect in the confined geometries.

Moreover, silicon is incorporated within molybdenum more rapidly than in the flat film, further promoting the formation of Mo–Si phases. This effect is not attributed to a variation in the intrinsic diffusion coefficient of Si in Mo but rather to the geometrical and morphological characteristics of the nanostructured catalyst. The nonplanar geometry introduces a higher density of interfaces, edges, and defect sites, which act as preferential pathways and trapping sites for diffusing species. As a result, the multidirectional diffusion regime enhances the probability of silicon incorporation within the molybdenum matrix compared with the predominantly unidirectional diffusion occurring in the flat film. This combination of carbon loss and enhanced silicon incorporation reduces the catalytic efficiency of nanostructured molybdenum. Furthermore, the reduced continuity and stability of the Mo_2_C phase in the nanostructured system can locally disrupt the catalytic activity associated with carbide formation, further affecting the graphene nucleation and growth. As a result, the multidirectional diffusion regime occurring in the nanostructured catalyst directly affects graphene growth, leading to a reduced number of graphene layers compared with the flat film. This effect is experimentally observed in the cross-sectional STEM analyses, where the flat sample exhibits approximately 15 graphene layers (Figure 2j), whereas the nanostructured sample shows only about 5 layers (Figure 6d).

In Figure 6b we can also observe the dewetting phenomenon that takes place during CVD, referring to the nanocircles (diameters of 100, 400, and 500 nm). Before the deposition, molybdenum nano and microstructures (circles and stars) are present on the TEOS surface. During chamber purging, conditioning, and pyrolysis reaction, solid-state dewetting occurs because of the high temperature (1020 °C), leading to the formation of small islands. As described before, the effect of dewetting is more evident for the smallest nanocircles (100 nm) because the molybdenum film is thinner than in nanocircles 2 and 3. A similar effect is observed for the four-pointed stars, in which the dewetting is more pronounced for the tip than the center of the stars (Figure 4d). These considerations are supported by cross-sectional TEM analyses. Figure 6c shows the islands (mean diameter 51.5 nm) formed in the nanocircles 2 (nominal diameter 400 nm) after dewetting for the nanostructured Graphene/Mo/TEOS/Si sample. Some molybdenum clusters are fully embedded within the TEOS substrate, while others are only partially incorporated due to the release of oxygen from the TEOS in gaseous form, which leaves voids in the matrix. Consequently, the reduction of exposed catalytic surface area limits the graphene formation. In the nanostructured samples, dewetting and geometrical confinement lead to a fragmented catalyst morphology, reducing the density of active catalytic sites compared to the flat film. At the same time, the increased density of interfaces promotes silicon incorporation and trapping within the molybdenum matrix, further enhancing catalyst contamination. These combined effects, together with multidirectional carbon diffusion, contribute to the reduced graphene thickness observed in the nanostructured samples.

## Conclusion

In this study, graphene growth by chemical vapor deposition was investigated on flat and nanostructured molybdenum films supported on TEOS/Si substrates. The high growth temperature (1020 °C) promotes interdiffusion among Si, Mo, and C and induces solid-state dewetting of nanostructured molybdenum. Under these conditions, metallic Mo is progressively transformed into molybdenum carbide (Mo_2_C), which represents the predominant phase after CVD and actively contributes to the catalytic process. In both systems, silicide and carbide phases form at the metal–oxide interface, highlighting the role of interfacial reactions and thermal compatibility. Structural analyses reveal that catalyst morphology critically affects graphene growth. In flat molybdenum films, carbon diffusion is predominantly unidirectional, enabling efficient surface segregation and the formation of multilayer graphene (∼15 layers). In contrast, nanostructured molybdenum exhibits multidirectional diffusion, leading to partial carbon loss, enhanced silicon incorporation, and reduced catalytic efficiency. Additional loss of exposed catalytic area due to dewetting further limits graphene growth to approximately 5 layers. Overall, these results demonstrate that surface morphology and interfacial diffusion, together with carbide formation, govern graphene nucleation and layer number on metal–oxide substrates. This mechanistic understanding provides a basis for controlling graphene growth through interface engineering and substrate design.

## Data Availability

Data will be made available on request.

## Abbreviations

CVD: Chemical Vapor Deposition
TEOS: Tetraethyl Orthosilicate
EBL: Electron Beam Lithography
RBS: Rutherford Backscattering Spectrometry
SIMS: Secondary Ion Mass Spectrometry
SEM: Scanning Electron Microscopy
TEM: Transmission Electron Microscopy
SAED: Selected Area Electron Diffraction
XRD: X-ray Diffraction
